# Cis-regulatory Function of the Pou5f1 Gene Promoter in the Mouse MHC Locus

**DOI:** 10.32607/actanaturae.27602

**Published:** 2025

**Authors:** V. V. Ermakova, E. V. Aleksandrova, A. A. Kuzmin, A. N. Tomilin

**Affiliations:** Institute of Cytology, Russian Academy of Sciences, St. Petersburg, 194064 Russia

**Keywords:** Pou5f1, Oct4, embryonic stem cells (ESCs), major histocompatibility complex, trophectoderm, regulation of gene expression

## Abstract

The *Pou5f1 *gene encodes the Oct4 protein, one of the key transcription factors required for maintaining the pluripotent state of epiblast cells and the viability of germ cells. However, functional genetics provides convincing evidence that *Pou5f1 *has a broader range of functions in mouse ontogeny, including suppression of atherosclerotic processes. Related studies have primarily focused on the functions of the Oct4 protein, while the regulatory sequences within the *Pou5f1 *gene have not been considered. In this study, we have developed a genetic model which is based on mouse embryonic stem cells (ESCs) for assessing the roles of the *Pou5f1 *gene promoter in the transcriptional regulation of neighboring genes within the major histocompatibility complex (MHC) locus. We have demonstrated that deletion of this promoter affects the expression of selected genes within this locus neither in ESCs nor in the trophoblast derivatives of these cells. A notable exception is the *Tcf19 *gene, which is upregulated upon *Pou5f1 *promoter deletion and might be associated with the atherosclerosis pathology due to its pro-inflammatory activity. The developed genetic model will pave the way for future studies into the functional contribution of the *cis*-regulatory association of *Pou5f1, Tcf19*, and, possibly, other genes with the atherosclerotic phenotype previously reported for mice carrying the* Pou5f1 *promoter deletion in vascular endothelial and smooth muscle cells.

## INTRODUCTION

The Oct4 protein, which is also known as a component of the Yamanaka cocktail and is used for the reprogramming of somatic cells into induced pluripotent stem cells (iPSCs), is among the key factors responsible for maintaining the pluripotent state of epiblast cells and their cultured analogs, embryonic stem cells (ESCs) [1]. ESCs and iPSCs, collectively referred to as pluripotent stem cells (PSCs), are capable of unlimited proliferation and differentiation into any type of somatic cells. The aforementioned properties make these cells a valuable tool for studying early embryogenesis,* in vitro *modeling of genetic diseases, and developing approaches in regenerative medicine. The self-maintenance and the choice of differentiation lineage of PSCs critically depend on Oct4 expression [2], with even slight changes in its levels having a significant effect on the fate of the PSCs [3, 4].

**Fig. 1:**
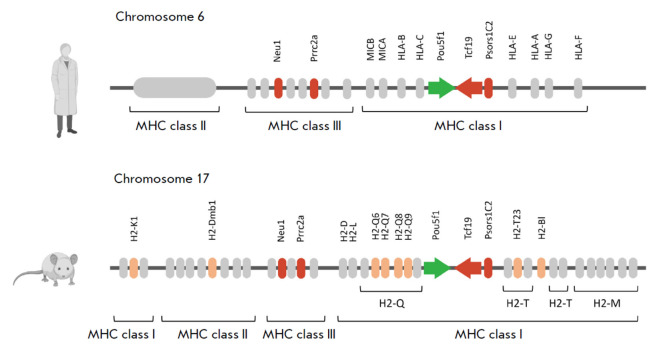
Schematic representation of the Pou5f1-MHC locus. A schematic depiction of the Pou5f1-MHC locus for human (top) and mouse (bottom). Genes analyzed in this study are highlighted: Pou5f1, in green; MHC genes, in orange; the genes potentially interacting with Pou5f1, including Tcf19, in red. The directions of transcription of the Pou5f1 and Tcf19 genes are additionally indicated with arrows. The figure was created using BioRender

The transcription factor Oct4 is encoded by the* Pou5f1 *gene, which resides within the major histocompatibility complex (MHC) gene cluster. The *Pou5f1* gene is located on the short arm of human chromosome 6 and on mouse chromosome 17 (*Fig. 1*). In both cases, this locus is among the most densely packed genomic regions [5] and comprises numerous genes encoding the proteins involved in the innate and adaptive immune responses and, particularly, those responsible for antigen processing and presentation [6].

Until today, it has been believed that a distal enhancer interacting with the *Pou5f1 *promoter in “naïve” PSCs, as well as a proximal enhancer being active in primed pluripotent cells, are sufficient to provide for the regulation of *Pou5f1 *expression and, therefore, proper functioning of PSCs and their proper exit from pluripotency [7, 8]. However, along with the classical regulatory elements of the *Pou5f1 *gene (the promoter, distal and proximal enhancers) described by Yeom et al. back in 1996 [9], advances in high-throughput sequencing techniques have led to the discovery of numerous, previously unknown *cis*regulatory elements that affect the expression of this gene [10, 11]. Hence, it has become clear that regulation of the *Pou5f1 *gene is a much more fine-tuned process than previously thought. To date, the specific roles of the individual regulatory elements involved in *Pou5f1 *expression control have been poorly characterized. Diao et al. demonstrated that just 17 out of the 41 identified regulatory elements of *Pou5f1 *serve as promoters for other protein-coding genes, including its nearest neighbor – *Tcf19 *[10]; however, it is unclear whether there is an opposite *cis*-regulatory association between *Pou5f1 *and the neighboring genes. Some findings showing a correlation between the risk of developing psoriasis and polymorphisms in the promoter region and the first exon of the *Pou5f1 *gene imply that there can be such an association [12].

An inverse correlation between *Pou5f1 *and *MHC* gene expression during ontogenesis has an interesting aspect. It is believed that in mouse ESCs, the expression level of MHC class I and II genes is low, while it increases during the differentiation of these cells [13, 14]. Meanwhile, according to the current paradigm, *Pou5f1 *expression is confined to PSCs and germ cells [9]. Therefore, it is possible that the protein- encoding activity of the *Pou5f1 *gene switches to the *cis*-regulatory one required to activate *MHC* genes. This mechanism is consistent with the findings in experiments on mice carrying a deletion of the *Pou5f1 *promoter region in smooth muscle and endothelial cells, which have shown a significantly deteriorated atherosclerotic phenotype, causing reduced plaque stability, lipid accumulation, inflammation, reduction of the mitochondrial membrane potential in endothelial cells, and decreased smooth muscle cell migration [15, 16].

In this study, we developed a genetic model that allowed us to assess the *cis*-regulatory function of the* Pou5f1 *promoter region with respect to the genes within the *Pou5f1-MHC *locus in ESCs and their differentiated progeny. Following a successful differentiation of ESCs into the trophoblast lineage via forced Cdx2 expression, we did not observe any regulatory role of the *Pou5f1 *promoter region in the expression of various genes within the MHC locus. However, we found that the *Pou5f1 *promoter represses the expression of the *Tcf19 *gene in both mouse ESCs and their trophoblastic derivatives.

## EXPERIMENTAL

**Obtaining mitotically inactivated embryonic fibroblasts**

Mouse embryonic fibroblasts (MEFs) were isolated in accordance with the current animal welfare laws of the Russian Federation, with approval from the Institute’s Ethics Committee (protocol No. 12/23).

MEFs derived from C57BL/6 mouse embryos (12-14 d.p.c.) were cultured on adhesive plastic pretreated with a 0.1% gelatin solution (Sigma, USA). The cells were cultured in a DMEM GlutaMAX medium (Gibco, USA) supplemented with 10% HyClone FBS (Cytiva, USA) and 1× penicillin/streptomycin (Gibco). After 4– passages, once a confluent cell monolayer had been formed, the MEFs were incubated for 2.5 h in a medium supplemented with 10 μg/mL mitomycin C (MMC, Sigma). After incubation, the cells were washed with PBS and cryopreserved for future use.

**Culturing of ESCs**

Mouse embryonic stem cells (ESCs) were cultured at 37°C in a humidified atmosphere containing 5% CO_2_ on plates for adherent cell cultures. A feeder layer of mitotically inactivated mouse embryonic fibroblasts (MMC-MEFs) with a density of 36 × 10³cells/cm² seeded into wells one day prior to the addition of ESCs, was used as a substrate. The cells were cultured in a standard S/L ESC medium containing KnockOut DMEM (Gibco) supplemented with 15% HyClone FBS (Cytiva), 1×NEAA (Gibco), 1× penicillin/ streptomycin (Gibco), 0.1 mM β-mercaptoethanol (Sigma-Aldrich), 2 mM L-glutamine (Gibco), and 1 : 5,000 in-house generated hLIF.

For reverting ESCs to the naïve pluripotent state, we used the 2i/L medium containing N2B27 (a mixture of DMEM/F12 (Gibco) and Neurobasal (1 : 1)) enriched with 1× N2, 1× B27 (without retinoic acid, Gibco), 50 μM β-mercaptoethanol (Sigma-Aldrich), 0.005% BSA (Sigma), 1× penicillin/streptomycin (Gibco), and 2 mM L-glutamine (Gibco) supplemented with 3 μM CHIR99021 (Axon), 1 μM PD0325901 (Axon), and 1 : 5,000 hLIF. The culture plates were pre-treated with a 0.01% poly-L-ornithine solution (Sigma).

**Plasmids**

The plasmid pRosa26-GOF-2APuro-MUT was constructed based on the plasmid Rosa26-GOF-2APuro described earlier [17]. pRosa26-GOF-2APuro-MUT carries a 9.8-kb fragment of the *Pou5f1 *gene, including its proximal and distal enhancers, homology arms targeting the *Rosa26 *locus, and a gene coding for resistance to a selectable marker, puromycin. A point synonymous mutation was introduced into the PAM sequence of the first exon of *Pou5f1 *within the plasmid pRosa26-GOF-2APuro to prevent knockout of exogenous* Pou5f1*.

The plasmid pRosa26-GR-Cdx2 carrying the Cdx2 sequence “fused” to the ligand-binding domain of the glucocorticoid receptor (GR) was ligated using constructs obtained earlier [18]. This plasmid also carries the gentamicin resistance gene and homology arms targeting the *Rosa26 *locus. A sequence of guide RNA (gRNA) 5’-ACTCCAGTCTTTCTAGAAGA-3’ paired with Cas9 nickase was used to incorporate the constructs into the alleles of the *Rosa26 *locus.

CRISPR/Cas9-mediated *Pou5f1 *knockout was performed using gRNA 5’- ACTCGTATGCGGGCGGACAT- 3’ encoded by the pX330-U6-Chimeric_ BB-CBh-hSpCas9-EGFP vector. The gRNA sequences were selected using Benchling, an online platform (www.benchling.com).

**Generating mutant ESC lines**

**Fig. 2:**
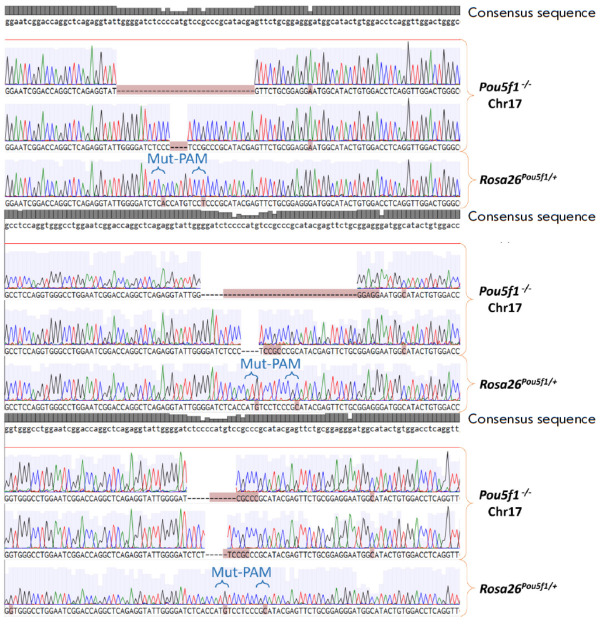
Sequences of endogenous Pou5f1 alleles from the Pou5f1-/-;Rosa26^Pou5f1/+^ cell line for three biological replicates. Note: “-/-” 1–3 – numbers of biological replicates for Pou5f1-/-;Rosa26^Pou5f1/+^ ESCs

In the first step of the generation of the *Pou5f1*-/-*; Rosa26Pou5f1*/*Cdx2 *ESC line, the *Pou5f1*+/+;*Rosa26Pou5f1*/+ line was used in order to produce cells with the* Pou5f1 *sequence placed in the *Rosa26 *locus and carrying a synonymous substitution within the first exon of *Pou5f1 *(the pRosa26-GOF-2APuro-MUT vector being utilized as a donor sequence). Next, to perform an endogenous *Pou5f1 *knockout, *Pou5f1*+/+;*Rosa26Pou5f1*/+ ESCs were transfected with the gRNA-/Cas9- encoding plasmid. Transfection was conducted using FuGene HD (Promega), in accordance with the manufacturer’s protocol. The knockout of endogenous* Pou5f1 *alleles and intact state of the exogenous construct within the *Rosa26 *locus were verified by Sanger sequencing of TA-cloned alleles (*Fig. 2*) alleles into the pAL2-T vector (Evrogen).

In order to generate *Pou5f1*-/-;*Rosa26Pou5f1*/*Cdx2 *and* Pou5f1*Δ/Δ;*Rosa26Pou5f1*/*Cdx2 *ESCs, the GR-Cdx2 sequence was incorporated into the second *Rosa26 *allele of the aforementioned ESC lines. The pRosa26-GR-Cdx2 vector was used as a donor sequence. Colonies were selected during six days using the geneticin antibiotic (G418) at a concentration of 500 µg/mL.

**Trophoblast differentiation**

The *Pou5f1*-/-; *R o s a 2 6Pou5f1*/ *C d x 2 *and *Pou5f1*Δ / Δ ;* Rosa26Pou5f1*/*Cdx2 *ESC lines were cultured in the S/L medium supplemented with G418 (500 μg/mL, Neofroxx) and puromycin antibiotics (1 µg/mL, Sigma-Aldrich). The cells were reverted to their naïve state by culturing under 2i/L conditions for 7 days and then passaged into wells coated with an MMC–MEF layer, then cultured in the TS medium based on a RPMI 1640 medium (Gibco) supplemented with 20% HyClone FBS (Cytiva), 1 mM sodium pyruvate (Gibco), 1× penicillin/streptomycin (Gibco), 0.1 mM β-mercaptoethanol (Sigma-Aldrich), 2 mM L-glutamine (Gibco), 1 μg/mL heparin (Hep) (Sigma- Aldrich), and 25 ng/mL fibroblast growth factor 4 (Fgf4) (Peprotech). The medium was pre-conditioned on MMC-MEFs for 72 h. A mixture of conditioned and fresh media at a 7 : 3 ratio was used for cell culturing. Dexamethasone (1 μM, Belmedpreparaty) and G418 (500 μg/mL, NeoFroxx) were added to the cells the next day after passaging. Four days later, the cells were reinoculated and cultured either in the standard TS medium or in the inflammation-mimicking TS medium. The latter was supplemented with either 300 U/mL interferon-gamma (IFNγ, ProSpec) or 1 μg/mL *E. coli *lipopolysaccharide (LPS, Sigma- Aldrich). Expression of trophoblast markers in the cells was analyzed one day after eliciting a pro-inflammatory response.

**Quantitative RT-PCR**

RNA was isolated using an RNA Solo kit (Evrogen); 1 μg of total RNA was utilized for cDNA synthesis. cDNA was synthesized in the presence of a RiboCare RNase inhibitor and MMLV reverse transcriptase (Evrogen). Real-time PCR was conducted on a LightCycler® 96 system (Roche) using 5× qPCRmix-HS SYBR (Evrogen). Primer specificity and the optimal annealing temperatures (*T*a) were pre-verified by PCR and electrophoresis using 4% agarose gel. *Table 1* lists the primer sequences and the selected *T*a values. The *GAPDH *housekeeping gene was utilized as a reference gene. At least three biological replicates and two technical replicates were used for each cell line.

**Table 1:** List of oligonucleotides used for quantitative real-time PCR

Primer	Nucleotide sequence 5’→3’	T, °C	Amplicon size, bp
qGAPDH-F	ACCCTTAAGAGGGATGCTGC	60	83
qGAPDH-R	CGGGACGAGGAAACACTCTC		
qOct4A-F	AGTGGAAAGCAACTCAGAGG	60	135
qOct4A-R	AACTGTTCTAGCTCCTTCTGC		
qCdx2-F	AGTCCCTAGGAAGCCAAGTGAA	60	96
qCdx2-R	AGTGAAACTCCTTCTCCAGCTC		
qCdx2GR-F	GCTGAAATCATCACCAATCAGATAC	60	134
qCdx2GR-R	CGCACGGAGCTAGGATACAT		
qCdx2endo-F	AGGCTGAGCCATGAGGAGTA	60	125
qCdx2endo-R	ctGAGGTCCATAATTCCACTCA		
qMash2-F	CGGGATCTGCACTCGAGGATT	65	86
qMash2-R	CCCCGTACCAGTCAAGGTGTG		
qTcfap2C-F	CGTCTCTCGTGGAAGGTGAAG	60	114
qTcfap2C-R	CCCCAAGATGTGGTCTCGTT		
qHand1-F	CCTACTTGATGGACGTGCTGG	60	129
qHand1-R	TTTCGGGCTGCTGAGGCAAC		
qElf5-F	CATTCGCTCGCAAGGTTACT	60	133
qElf5-R	GAGGCTTGTTCGGCTGTGA		
qH2-K1-F	TCCACTGTCTCCAACATGGC	60	113
qH2-K1-R	CCACCTGTGTTTCTCCTTCTCA		
qH2-Q6,8-F	CTGACCCTGATCGAGACCCG	60	112
qH2-Q6,8-R	TGTCCACGTAGCCGACGATAA		
qH2-Q7,9-F	GAGCTGTGGTGGCTTTTGTG	68	85
qH2-Q7,9-R	TGTCTTCATGCTGGAGCTGG		
qH2-Q10-F	ACATTGCTGATCTGCTGTGGC	60	120
qH2-Q10-R	GTCAGGTGTCTTCACACTGGAG		
qH2-Dmb1-F	ATGGCGCAAGTCTCATTCCT	68	95
qH2-Dmb1-R	TCTCCTTGGTTCCGGGTTCT		
qH2-Bl-F	ACCGGCTCCAACATGGTAAA	60	114
qH2-Bl-R	AGGAAGGATGGCTATTTTTCTGCT		
qH2-T23-F	ATAGATACCTACGGCTGGGAAATG	60	105
qH2-T23-R	AGCACCTCAGGGTGACTTCAT		
qTcf19-F	GATGATGAGGTCTCCCCAGG	60	107
qTcf19-R	TTTCCCTGTGGTCATTCCCC		
qPsors1C2-F	CTGTGTGCAGGAGGCATTTC	68	86
qPsors1C2-R	AGGGATCACCAGGGATTGGG		
Gm32362-F	GTCTGGAGAACCAAAGACAGCA	60	114
Gm32362-R	TTACAGCTTGGGATGCTCTTC		
Prrc2a-F	GAGATCCAGAAACCCGCTGTT	60	104
Prrc2a-F	TTCAGGCTTGGAAGGTTGGC		
Neu1-F	CCGGGATGTGACCTTCGAC	60	127
Neu1-R	CAGGGTCAGGTTCACTCGGA		
TNF-F	GTGCCTATGTCTCAGCCTCTT	60	117
TNF-R	AGGCCATTTGGGAACTTCTCATC		

## RESULTS

**Generation of control *Pou5f1 *knockout ESC lines**

In order to investigate the *cis*-regulatory role of the* Pou5f1 *promoter region in ESCs and their differentiated derivatives, we used the previously generated ESC line carrying a Cre-mediated deletion of the *loxP*-flanked promoter and the first exon of the* Pou5f1 *gene. These cells maintain pluripotency owing to the expression of an exogenous *Pou5f1 *fragment inserted into the *Rosa26 *locus (*Pou5f1*Δ/Δ;*Rosa26Pou5f1/+*) [17]. The deletion in this cell line is identical to that introduced when studying the role of the transcription factor Oct4 in mouse cellular models of atherosclerosis (smooth muscle and endothelial cells) [15, 16]. We complemented this cell line with a new control line, *Pou5f1*-/-;*Rosa26Pou5f1*/*+*, where endogenous* Pou5f1 *had been knocked out via indel mutations in the first exon. Like for the *Pou5f1*Δ/Δ;*Rosa26Pou5f1*/+ cell line, Oct4 expression was maintained via a 9.8-kb* Pou5f1 *fragment inserted into one of the *Rosa26 *alleles (*Fig. 2A*). This approach helped to eliminate the variability of Oct4 expression between the two ESC lines. This variability would inevitably arise when using the *Pou5f1*Δ/+ cell line. Importantly, the *Pou5f1- *allele had retained an intact promoter, making it possible to compare its functions directly with those of the *Pou5f1*Δ allele. Previously, we have found that the *Rosa26Pou5f1 *allele can ensure self-maintenance of* Pou5f1*Δ/Δ;*Rosa26Pou5f1*/+ ESCs; however, these cells are unable to differentiate properly because the 9.8-kb* Pou5f1 *fragment lacks all the essential *cis-*regulatory elements responsible for proper gene regulation during differentiation [17]. Therefore, directed differentiation of *Pou5f1*Δ/Δ;*Rosa26Pou5f1*/+ and *Pou5f1*-/-;* Rosa26Pou5f1*/+ ESCs represented a separate problem that needed to be addressed in this study.

**Assessment of the ability of generated ESCs to differentiate into the trophoblast lineage**

We chose the trophoblast lineage to differentiate ESCs into. It is known that trophoblast cells, which ultimately segregate at the late blastocyst stage as trophectoderm, endow maternal immune tolerance to the fetus after implantation by actively synthesizing non-classical MHC molecules [19]. Furthermore, trophoblast segregation is accompanied by *Pou5f1* silencing [20], which may trigger promoter activity switch from regulating *Pou5f1 *itself to regulating the neighboring MHC-cluster genes [21]. Therefore, we concluded that trophoblast differentiation may serve as a suitable model for assessing gene expression profiles within the *Pou5f1-MHC *locus.

**Fig. 3:**
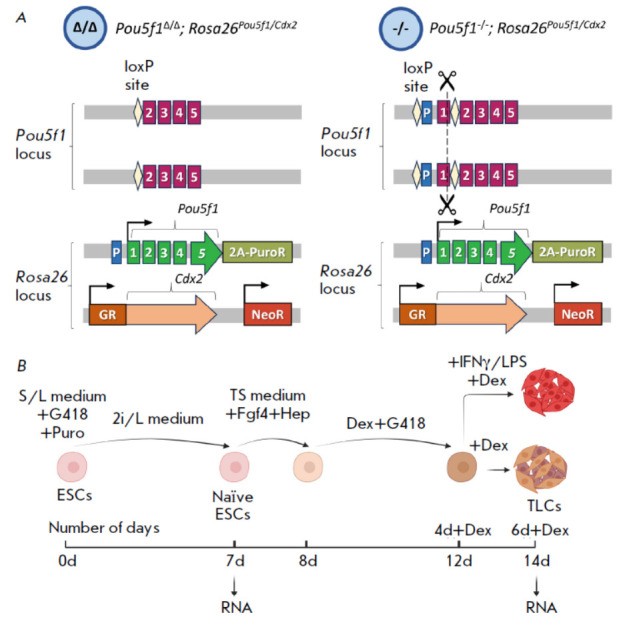
Cell lines and the experimental protocol. (A) Schematic representation of the experimental embryonic stem cell (ESC) lines. “Δ/Δ” – Pou5f1Δ/Δ; Rosa26Pou5f1/Cdx2 ESC line with a deletion of the endogenous Pou5f1 promoter; “-/-” – Pou5f1-/-;Rosa26Pou5f1/Cdx2 ESC line with an intact endogenous promoter and an inactivating indel mutation in the first exon of the gene. P – promoter; 1–5 – exons of the Pou5f1 gene; 2A-PuroR – P2A site followed by the puromycin resistance gene PuroR; GR – ligand-binding domain of the glucocorticoid receptor; NeoR – the G418/neomycin resistance gene. (B) Schematic representation of ESC differentiation towards the trophoblast lineage (see the Materials and Methods section for a detailed description). Fgf4 – fibroblast growth factor 4; Hep – heparin; Dex – dexamethasone; IFNγ – interferon gamma; LPS – lipopolysaccharides; TLCs – trophoblast-like cells. The figure was created using BioRender

The differentiation protocol was based on forced expression of Cdx2, a key master regulator of trophoblast development [22, 23], which was also inserted into the *Rosa26 *locus. The approach was chosen as the most straightforward alternative to those relying on media and growth factors, owing to its simplicity and the available published protocols. For controlled trophoblast differentiation, we used Cdx2 as a component of the fusion protein containing a ligand-binding domain of a glucocorticoid receptor (GR) that was activated by adding dexamethasone (Dex) to the medium. *Figure 3A* shows the final configurations of the* Pou5f1*Δ/Δ;*Rosa26Pou5f1*/*Cdx2 *and *Pou5f1*-/-;*Rosa26Pou5f1*/*Cdx2* ESC lines.

Since the efficiency of trophoblast differentiation of ESCs under forced Cdx2 expression depends on the pluripotent stage [24], at the initial differentiation stage, *Pou5f1*Δ/Δ;*Rosa26Pou5f1*/*Cdx2 *and *Pou5f1*-/-;* Rosa26Pou5f1*/*Cdx2 *ESCs were reverted to their naïve state by 7-day culturing in the 2i/L medium. Furthermore, this experimental timepoint was used for monitoring changes in gene expression over time. The second and hinge study point was on Day 6 of cell culturing in the presence of dexamethasone, corresponding to Day 14 of the entire experiment (*Fig. 3B*).

**Fig. 4:**
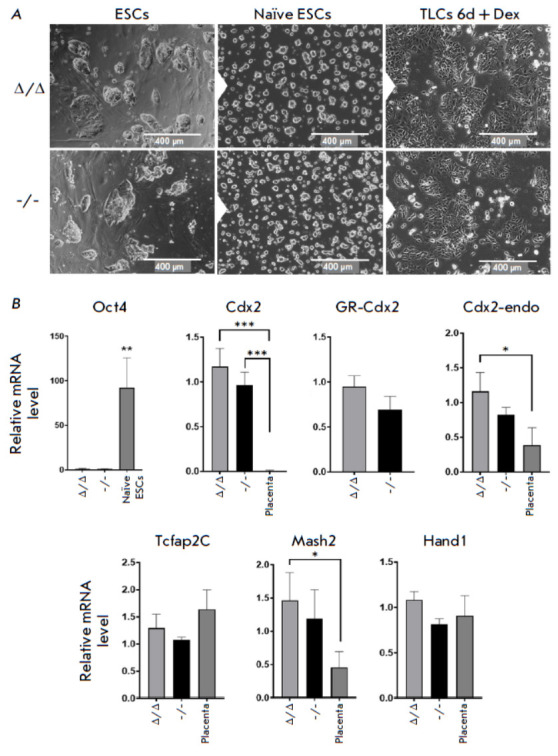
Validation of the ability of Pou5f1Δ/Δ;Rosa26Pou5f1/Cdx2 and Pou5f1-/-;Rosa26Pou5f1/Cdx2 ESC lines to differentiate towards the trophoblast lineage. (A) Morphological characteristics of cells at different stages of differentiation: serum (S/L) culture conditions (left), naïve (2i/L) culture conditions (middle), and trophectoderm cells induced by Dex treatment for six days (right). (B) Analysis of the expression of trophoblast markers (Cdx2, Tcfap2C, Mash2, and Hand1) during differentiation compared to placenta. Designations are the same as those in Fig. 3A. *P ≤ 0.05; **P ≤ 0.01; ***P ≤ 0.001 according to ANOVA

By Day 6 of culturing in the presence of Dex, the cells, which originally had had a dome-shaped (under the SL conditions) or spherical (under the naïve 2iL conditions) colony shape, had morphed into flat colonies with clearly defined borders and an angular cell morphology, resembling those previously described for trophoblast stem cells [22, 23] (*Fig. 4A*).

An analysis of the marker expression profile on Day 6 of differentiation in the presence of Dex revealed a significant decline in the Oct4 mRNA level (compared to that in naïve ESCs) and an increase in the levels of trophectoderm marker mRNA in both cell lines. Mouse placenta was used as a control for the expression levels of trophoblast markers. The total Cdx2 levels in both ESC lines were significantly higher than that in the placenta. Differential analysis of endogenous Cdx2 and exogenous GR-Cdx2 mRNA levels established that this difference in the total Cdx2 levels was due to an induced overexpression of GR-Cdx2. Meanwhile, the endogenous Cdx2 level also increased to a level akin to that in placenta. We revealed no statistically significant differences in Cdx2 expression between the

**Assessment of the impact of the *Pou5f1* promoter region on gene expression within the *Pou5f1-MHC *locus**

**Fig. 5:**
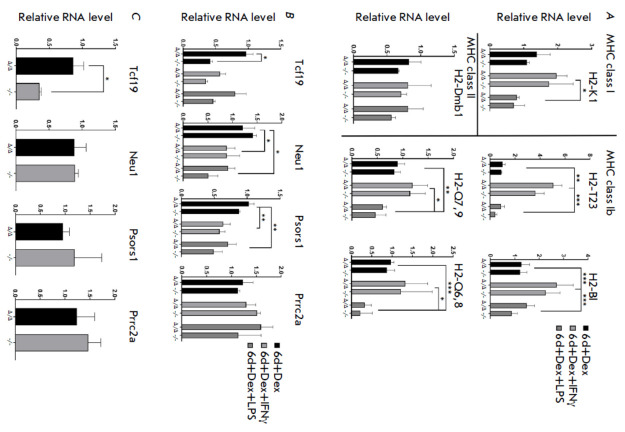
Comparison of Pou5f1-MHC locus-related gene expression between the Pou5f1Δ/Δ;Rosa26Pou5f1/Cdx2 and Pou5f1-/-;Rosa26Pou5f1/Cdx2 cell lines under standard and pro-inflammatory culture conditions. (A, B) Comparison of the relative mRNA levels between the Pou5f1Δ/Δ;Rosa26Pou5f1/Cdx2 and Pou5f1-/-;Rosa26Pou5f1/Cdx2 ESC lines after six days of culture with dexamethasone (Dex) under standard and pro-inflammatory conditions (with IFNγ or LPS). Panel (A) presents the expression analysis of MHC class I and II genes; panel (B) compares the expression of the genes within the Pou5f1-MHC locus that were previously demonstrated to exhibit cis-regulatory activity towards Pou5f1. (C) Comparison of the expression of the genes from panel (B) in undifferentiated Pou5f1Δ/Δ;Rosa26Pou5f1/Cdx2 and Pou5f1-/-;Rosa26Pou5f1/Cdx2 ESCs cultured under 2i/L conditions. Figure legend is the same as that in Fig. 3A. *P ≤ 0.05; **P ≤ 0.01; ***P ≤ 0.001 according to ANOVA. Comparisons were performed between the “Δ/Δ” and “-/-” cell lines under each culture condition, as well as between different conditions using the Tukey’s test

During the experiment, the cells were divided into groups and exposed to IFNγ or lipopolysaccharide (LPS). IFNγ and LPS are commonly utilized in various* in vitro *and *in vivo *inflammation models, so we addressed the hypothesis holding that induction of pro-inflammatory signals would promote the upregulation of the expression of immune-related genes, including the MHC genes, which would allow to more thoroughly assess the differences in the expression of the selected genes between generated cell lines. However, the differences in the expression of several MHC genes (*H2-K1, H2-T23, H2-Bl, H2-Dmb1, H2-Q6,8*, and *H2-Q7,9*) had been induced already by culture conditions, while their expression levels were identical in the *Pou5f1*Δ/Δ;*Rosa26Pou5f1*/*Cdx2 *and* Pou5f1*-/-;*Rosa26Pou5f1*/*Cdx2 *ESCs (*Fig. 5A*). *Tcf19 *was the only gene whose expression was significantly different between the two genotypes (*Fig. 5B*). Notably, in undifferentiated *Pou5f1*Δ/Δ;*Rosa26Pou5f1*/*Cdx2 *ESCs cultured under naïve (2i/L) conditions, Tcf19 expression was already elevated compared to that of* Pou5f1*-/-;*Rosa26Pou5f1*/*Cdx2 *ESCs (*Fig. 5C*).

## DISCUSSION

The question regarding the existence of *Pou5f1 *expression outside the generally accepted concept of pluripotency remains to be addressed. The available evidence suggests that *Pou5f1 *plays no functional role in differentiated mammalian cells, as indicated by the absence of phenotypic effects to the knockout of this gene and potential errors in the interpretation of the immunostaining and RT-PCR data [25, 26, 27]. On the other hand, recent research using functional genetic approaches convincingly demonstrates the role played by *Pou5f1 *in somatic cells. Among those, there are studies describing the effect of *Pou5f1 *knockout in smooth muscle and endothelial cells, as well as the study by Zalc et al., who had revealed *Pou5f1 *reactivation in cranial neural crest cells and substantiated its role in enhancing the differentiation potential of these cells during embryogenesis [15, 16, 28].

Our hypothesis could integrate the reported findings from the perspective of the *cis*-regulatory properties of the *Pou5f1 *promoter, confirming the activity of this gene on the one hand, while, on the other hand, decoupling it from the Oct4 protein, the product of this gene.

Elucidating the precise mechanism of how the* Pou5f1 *gene functions in the context of atheroscle rosis is a critical endeavor whose resolution is of certain importance not only for fundamental research, but also for potential medical applications. Thus, if the effects reported for atherosclerosis models have anything to do with the transcription factor Oct4, it should be regarded as a potential effector protein in the therapy of this disease. If the atherosclerotic phenotype is related to the *cis*-regulatory activity of the* Pou5f1 *promoter, the focus of therapeutic strategies should be shifted toward the modulation of this activity.

Unlike the approach presented in this work, the earlier models for studying the *Pou5f1 *gene were primarily designed to investigate its function in pluripotent stem cells, and the pluripotency of the cells was maintained using transgenic *Pou5f1 *cDNA under the control of constitutive promoters [3, 29]. Not only did our approach allow us to generate an isogenic pair of cell lines with *Pou5f1 *expression inactivated during directed differentiation, but it also made it possible to compare them because of the identical location of exogenous *Pou5f1*, which would have been impossible if lentiviral vectors had been used. We believe that the developed model can help answer the question regarding *Pou5f1 *expression in differentiated cells. The present study is the first step towards doing that. Although we did not observe any sweeping effect of* Pou5f1 *promoter deletion on the expression of the genes within the *MHC *locus, one of the studied genes,* Tcf19*, was found to be susceptible to the introduced modifications. Interestingly, this gene is the nearest neighbor of *Pou5f1*, which may facilitate the interplay between their regulatory sequences. On the other hand, since the observed differences between the cell lines arise at the pluripotent stage, the mechanistic scenario for the effect of the introduced deletion can be considered definitely plausible. Thus, in the case of competition between the transcriptional machineries of the oppositely oriented *Tcf19 *and *Pou5f1 *genes, deletion of the *Pou5f1 *promoter may relieve transcriptional interference, thereby favoring the expression of* Tcf19*. Although such a highly specific effect was unexpected, it appears to be consistent with the central concept of pluripotency. Being transcriptionally active in pluripotent cells, *Pou5f1 *may, through alterations in its activity (e.g., due to specific mutations), affect the expression of *Tcf19*, potentially initiating a cascade of gene regulatory disruptions in daughter cells, including non-pluripotent ones. In turn, it may contribute to the development of various pathologies. This hypothesis offers a plausible explanation for the findings obtained in studies that have focused on *Pou5f1* polymorphisms associated with psoriasis [12], especially taking into account the association between *Tcf19* and this disease [30, 31]. Interestingly, *Tcf19 *may also be involved in inflammatory responses, thus linking our findings to the data obtained using atherosclerosis models [32, 33]. A point of difference lies in the fact that *Pou5f1 *knockout in those models was conditional; i.e., it was induced specifically in vascular smooth muscle or endothelial cells. Nonetheless, it remains possible that even the deletion of a methylated* Pou5f1 *region could enhance *Tcf19 *expression, which requires further investigation.

## CONCLUSIONS

In this study, we developed a unique genetic model for investigating the role of the *Pou5f1 *promoter sequence in the regulation of the expression of the genes that do not play a crucial role in pluripotent cells, providing a tool for uncovering potential non-classical functions of *Pou5f1 *in differentiated cells. We have partially confirmed the hypothesis on the *cis*-regulatory activity of the *Pou5f1 *promoter region with respect to the genes residing within the* Pou5f1*-*MHC *locus (to be more precise, with respect to its nearest neighbor, the *Tcf19 *gene). Future research will focus on refining the regulatory landscape of the *Pou5f1*-*MHC *locus in other types of differentiated cells.

## Abbreviations

iPSC: induced pluripotent stem cells
TLCs: trophoblast-like cells
ESCs: embryonic stem cells
MEFs: mouse embryonic fibroblasts
MMC: mitomycin C
Fgf4: fibroblast growth factor 4
IFNγ: interferon γ
LPS: lipopolysaccharide
MHC: major histocompatibility complex
gRNA: guide RNA
GR: glucocorticoid receptor
